# Supplementary material for: Interactome Profiling of N-Terminus-Truncated NS1 Protein of Influenza A Virus Reveals Role of 14-3-3γ in Virus Replication
Source: Pathogens. 2022 Jun 27;11(7):733. doi: 10.3390/pathogens11070733 (PMC9321973; doi:10.3390/pathogens11070733)
Supplement: Supplementary file 1 [file pathogens-11-00733-s001.zip › Supplemental Figure S1.pdf]

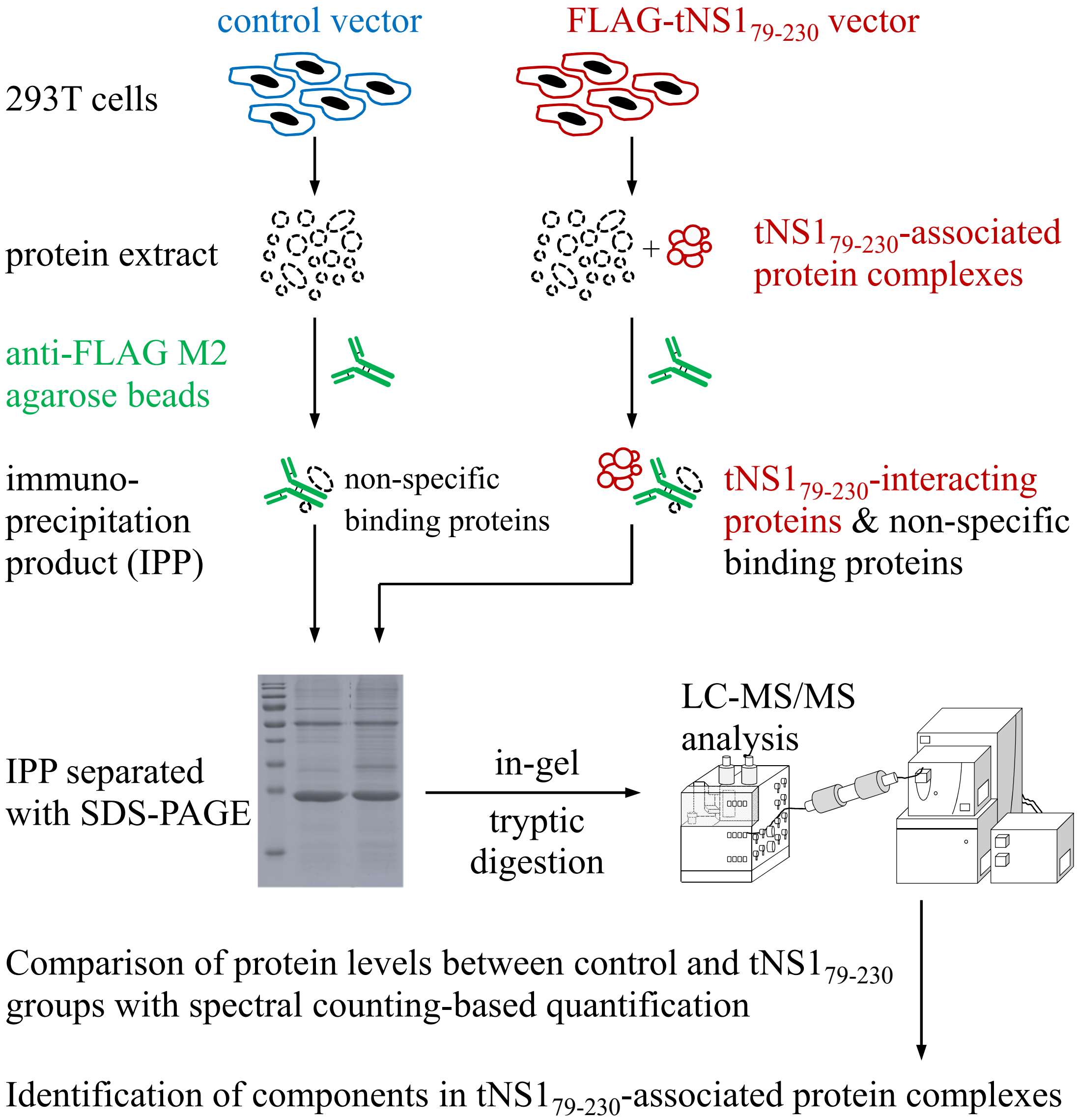

Supplemental Figure S1. Workflow for identification of components in the tNS1$_{79-230}$-associated protein complexes. To explore cellular proteins that interact with influenza tNS1$_{79-230}$ protein, 293T cells were transfected with a control vector and plasmid that expresses 3xFLAG-tagged tNS1$_{79-230}$ encoded by influenza A virus PR8(H1N1) strain. After 24 h, lysates of the transfected cells were subjected to immunoprecipitation (IP) with anti-FLAG resin. The IP products of control and tNS1$_{79-230}$ groups were separated with 10% SDS-PAGE and stained with Colloidal Blue Staining kit. Each gel lane was then divided into 20 fractions. After in-gel digestion with trypsin, peptides from each gel fraction were analyzed by LC-MS/MS for triplicates. Spectral searches were performed with Mascot Server in a Swiss-Prot database and results were further integrated with the Scaffold software. With the spectral counting-based label-free quantification approach, protein levels between the control and tNS1$_{79-230}$ groups were compared. The fold change of each protein was determined by dividing average spectral count of the protein in the tNS1$_{79-230}$ group by that in the control group. Mean and standard deviation (SD) of the fold changes of all identified proteins were determined. A protein with a fold change larger than the mean plus two SD were deemed as a candidate actually involved in tNS1$_{79-230}$-associated protein complexes.
