# Supplementary material for: Interactome Profiling of N-Terminus-Truncated NS1 Protein of Influenza A Virus Reveals Role of 14-3-3γ in Virus Replication
Source: Pathogens. 2022 Jun 27;11(7):733. doi: 10.3390/pathogens11070733 (PMC9321973; doi:10.3390/pathogens11070733)
Supplement: Supplementary file 1 [file pathogens-11-00733-s001.zip › Supplemental Table S4.pdf]

Supplemental Table S4. Components identified in protein complexes of full-length and truncated NS1 proteins[a]

Protein group	Protein number	Protein list
Proteins identified in both analyses	10	FLG2, IMPDH2, CCT2, MDH2, DDX47, ECHDC1, PNO1, YWHAG, NSA2, DDX39B
Proteins uniquely detected in full-length NS1 interactome analysis	54	HNRNPA3, ANXA2, PRPF19, CSNK2A1, FIBP, NCOA5, YWHAQ, HSPA4, SNRNP40, BRIX1, SNRPA1, YWHAH, PSMD6, PRKRA, RBMXL1, XAB2, STAU1, DHX8, DIAPH1, PTBP1, C1QBP, SEPTIN2, YTHDC2, RPRD1A, AHCTF1, DNAJB6, HSPBP1, CPVL, RPL7L1, CIT, IGF2BP2, QPCT, SAFB2, SAFB, FANCI, NIFK, MSI1, PIK3R2, FANCD2, ISY1, C1orf35, TUBA1A, DNAJB11, PABPN1, MBOAT7, HTT, YWHAB, CPOX, DYNC2H1, YWHAE, AKR1B1, NMNAT1, AUP1, ZFR
Proteins exclusively identified in tNS1 interactome analysis	36	RPS11, HIST1H2BC, RPL12, PPL, HSPB1, MYO1B, DHRS4, TRNAU1AP, LGALS7B, SFN, RPL26, HIST1H2AD, EVPL, PPIA, LMNA, CASP14, KPRP, TRIM29, EPPK1, DSG1, RPL28, CALML5, ZC3HAV1L, SERPINB3, FLG, HAGH, AHNAK, DSP, LIG3, TUBA1C, STK38, LONP1, COQ5, SBSN, POF1B, IVL

[a] The information of proteins interacted with the full-length NS1 is adopted from Kuo et al., J Proteome Res. 2016, 15(5), 1639-48. The proteins listed in Table 1 are considered as the tNS1-interacting partners.
